# RNA Regulatory Networks as a Control of Stochasticity in Biological Systems

**DOI:** 10.3389/fgene.2019.00403

**Published:** 2019-05-07

**Authors:** Marylène Vandevenne, Michael Delmarcelle, Moreno Galleni

**Affiliations:** InBioS – Center for Protein Engineering, University of Liège, Liège, Belgium

**Keywords:** regulatory RNA networks, non-coding RNAs, cell stochasticity/determinism, RNA world theories, origins of life

## Abstract

The discovery that the non-protein coding part of human genome, dismissed as “junk DNA,” is actively transcripted and carries out crucial functions is probably one of the most important discoveries of the past decades. These transcripts are becoming the rising stars of modern biology. In this review, we have casted a new light on RNAs. We have placed these molecules in the context of life origins, evolution with a big emphasize on the “RNA networks” concept. We discuss how this view can help us to understand the global role of RNA networks in modern cells, and can change our perception of the cell biology and therapy. Finally, although high-throughput methods as well as traditional case-to-case studies have laid the groundwork for our current knowledge of transcriptomes, we would like to discuss new strategies that are better suited to uncover and tackle these integrated and complex RNA networks.

## Introduction: Non-Coding RNA in the Spotlights

Proteins have been for a long time considered as major effectors of most cellular processes involved in cell metabolism, homeostasis, and genetic regulation. If DNA had the second role of genetic information storage, RNA was reduced to a simple genetic intermediate step between DNA and proteins. It’s only recently that the scientific community has gain increasing interests in RNAs for several observations. One of the most intriguing one is the contradiction between the number of protein-coding genes and the complexity of organisms. For example, the size of the human genome is currently estimated to 19 000 protein-coding genes (Ezkurdia et al., 2014) which is far below the 100 000 genes that were initially predicted. These 19 000 coding genes are unexpectedly slightly fewer compared to those of nematodes (*Caenorhabditis elegans* (Hillier et al., 2005) with ≈ 20 000 protein-coding genes or flies (*Drosophila melanogaster*) with 14 000 protein-coding genes and only five fold more abundant compared to bacteria (*Escherichia coli*) with ≈ 4 500 protein-coding genes. The source of organism complexity and diversity might therefore rather rely on how these genes are used and regulated (King and Wilson, 1975; Franchini and Pollard, 2017). A second important observation was highlighted in 2012, when the international ENCODE project has established for the first time that 75% of the human genome is transcripted into RNAs while only 2% of these transcripts are translated into proteins (ENCODE Project Consortium, 2012). This indicates that 98% of the transcripts are not translated into proteins. Therefore RNAs are much more abundantly represented in the cell compared to proteins.

The last past years have witnessed a strong interest in these, so-called, non-coding RNAs (ncRNAs), since many of them have emerged as key players in the cell biology with important regulatory roles and, therefore, were associated to numerous diseases ranging from cancers to neurological disorders (Taft et al., 2010; Fu, 2014; Barta and Jantsch, 2017). Thus, ncRNAs represents a gold mine of potential new biomarkers and drug targets. However, we have only started to scratch the surface of regulatory ncRNAs. Their structures, precise molecular mechanisms, biological functions and overall role of this huge amount of RNAs in the cell remains poorly understood.

Importantly, if various classes of regulatory RNAs (e.g., miRNAs, siRNAs, …) are single-stranded and act by base pairing with other nucleic acids (RNAs or DNAs), it is very likely that a vast majority of non-coding transcripts adopts complex 3D structure(s) to achieve their biological functions. These “structured” RNAs act using very diverse mechanisms including RNA-RNA, RNA-ligand, RNA-protein, RNA-DNA, and RNA-substrate interactions (Wang and Chang, 2011). However, currently, less than 1% of all the structures reported in the Protein Data Bank (PDB) are RNA structures. Like proteins, RNA structures have different organization levels: the first one consists in the nucleotide sequence that folds on itself via Watson–Crick base-pairing to form secondary structure elements (e.g., hairpins, bulges…) and unpaired regions. Finally, these elements are precisely organized in space to form the tertiary structure of the RNA that is, in most cases, stabilized by divalent ions, e.g., Mg^2+^ (Westhof, 2000). Finally, it is worth to mention that RNA structures are highly dynamic and modulated by binding to partners, which add another degree of complexity to these structures.

## The “RNA World” and RNA Networks Theories of Life Origins

The molecular mechanisms and actors that have led to the origin of life billions of years ago remain among the most fundamental unsolved enigmas of modern science. In this context, RNAs were suggested to be the first biological molecules on Earth, mostly because of their ability to do both: store genetic information and catalyze various biochemical reactions (Yanagawa, 1994; Higgs and Lehman, 2015). In addition, RNA has different properties that make it the ideal candidate as the predecessor of proteins and DNA: (i) it can exists in a single-stranded form, in duplexes or adopt more complex structures; (ii) RNA subunits (e.g., ATP) constitute a source of energy; (iii) and finally RNA has the ability to evolve under selective pressure, as demonstrated in SELEX experiments (Systematic Evolution of Ligands by Exponential enrichment) (Alberts et al., 2002; Harris, 2010; Yarus, 2010). Finally, self-replicating RNAs have been developed *in vitro* (Ekland and Bartel, 1996; Johnston et al., 2001; Shechner and Bartel, 2011; Robertson and Joyce, 2014) using engineered ribozymes (catalytic RNAs) with RNA-template RNA ligase activities that join oligonucleotide substrates to form complementary RNA products (James and Ellington, 1999; Levy and Ellington, 2001; Robertson and Joyce, 2014). This multitask property and high plasticity in terms of structures and activities of RNAs strongly support the hypothesis of the, so-called, “RNA world” theory. According to this hypothesis, it’s only later in evolutionary time that DNA arose and took over the storage of genetic information whereas proteins supported the catalysis tasks in the cells. Therefore, it is reasonable to state that life has started with non-specialized molecules (RNAs) able to accomplish different tasks but with limited efficiencies. Evolution has led to the selection of more specialized molecules (DNA, proteins) able to take over restricted functions in the cells but with much higher efficiencies. Indeed, catalytic RNAs increase reaction rates by up to 10^11^-fold with reaction efficiencies (*k*_cat_/*K*_m_) up to 10^8^ M^-1^ min^-1^, which is 10^3^-fold less than what is observed for proteins catalyzing equivalent reactions (Cech, 1993; Narlikar and Herschlag, 1997; Tanner, 1999). Thus, compared to proteins and DNA, RNAs can be seen as the most fundamental elements in the cell which explains why, nowadays, RNAs are found in all fundamental processes in the cell (tRNA, rRNA, and mRNA…).

Besides the simplified and “individualistic” view of a unique auto-replicative RNA molecule at the origins of life, another theory, rather “communistic,” postulated that life started with ensembles of RNA molecules (Yeates and Nehman, 2016). This theory is seducing, because the definition of “life” consists in an ensemble of physical entities that carry out biological processes, and form a system that is self-sustaining and capable of Darwinian evolution. Therefore, this communistic view of life origins where a self-sustaining system arose from different populations of RNAs that interact with each other and have complementary tasks to manage different processes (ex: catalysis, support for genetic information, and substrates/products of chemical reactions) is an hypothesis that has been well admitted by the scientific community (Eigen and Schuster, 1977; Ganti, 2003; Vaidya et al., 2012; Vasas et al., 2012; Hordijk and Steel, 2013; Higgs and Lehman, 2015; Yeates and Nehman, 2016). In this “*RNA network*” hypothesis, each individual RNA harbors one or several function(s) that complement(s), or partially overlap(s) with the functions carried out by other RNAs. This concept of prebiotic networks constituted of interacting RNA species that evolve and act co-operatively has been demonstrated experimentally (Vaidya et al., 2012) and mathematically modeled (Hordijk and Steel, 2013). Indeed, co-operating molecules with complementary activities make the biological system more robust to external and internal changes.

Notably, the “individualistic” and “communistic” theories are not contradictory. We can imagine that the first theory evolves toward the second one, and similarly, the second one evolves toward the first one to give auto-replicative entities. However, the communistic theory is surely more admitted by the scientific community. First, co-operative replication within these ensembles of RNAs is easier than self-replication of a single RNA (Kauffman, 1993). Secondly, cooperative molecular networks have demonstrated fitness benefits and selection preferences compared to selfish entities (Eigen and Schuster, 1977; Ganti, 2003; Vaidya et al., 2012; Vasas et al., 2012; Hordijk and Steel, 2013; Higgs and Lehman, 2015).

## Understanding the Global Roles of RNAs in the Cell Biology

It is interesting to note that, in response to specific nutritional or environmental conditions, all the cell signaling pathways starting from stimuli perception, activation of appropriate genes and then modification of the cell behavior have been described with proteins as main actors of cell decisions and homeostasis. If this is a valid simplification in prokaryotic cells, where only 12% of prokaryotic genomes are non-protein coding DNA (Ahnert et al., 2008). This cannot be true for eukaryotic cells where the percentage of non-protein coding DNA increases quadratically with organism complexity (Ahnert et al., 2008) to reach 98% in human (Mattick, 2001). This huge energetic cost associated with massive transcription of the genome cannot be due to random or residual RNA polymerases activities: it has to have an important purpose for the cell biology that proteins and DNA are not able to carry out. An increasing number of evidences show that besides housekeeping functions, numerous RNAs carry out important regulatory roles in both eukaryotic and also in prokaryotic cells using highly diverse mechanisms [for reviews on eukaryotic and prokaryotic mechanisms of RNAs (see Waters and Storz, 2009; Marchese et al., 2017)]. This high diversity in RNA mechanisms is directly associated with their high plasticity in terms of structures, partners of interaction and therefore functions (Ancel and Fontana, 2000). However, it is interesting to note that, so far, we don’t really have yet a comprehensive and overall understanding of the global roles of RNA networks in the cell and how RNA and protein networks are integrated to regulate gene expression and cell fate.

**FIGURE 1:**
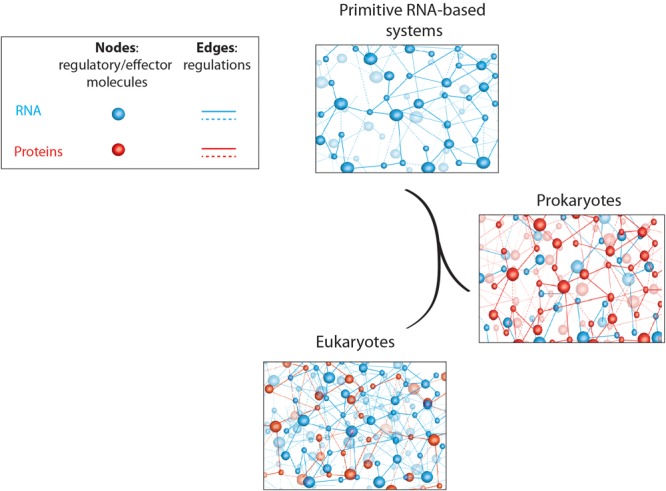
Schematic representation of primitive and modern cell regulatory networks of interacting molecules. RNA- and protein-based networks are represented in blue and red, respectively. Edges (lines) represent interactions/regulations between nodes (circles) that correspond to regulatory or effector molecules (proteins or RNA). This figure highlights the relative abundance of coding (proteins) and non-coding (RNAs) regulatory elements in living organisms; starting with “rudimentary” RNA-based networks in “primitive” systems to complex and dense RNA networks in higher eukaryotic organisms whereas prokaryotes rather use protein-based regulatory networks.

Based on the observations described above, it seems that prokaryotes encode a large majority of their regulatory overheads in proteins, whereas eukaryotes rather recruit regulatory RNAs for this purpose (Pheasant and Mattick, 2007; Taft et al., 2007; Ahnert et al., 2008). This observation is somehow surprising if we consider the RNA world theories discussed in this review. Indeed, modern cells evolved from the most primitive life form, which presumably consisted in organized RNA networks, and gave rise to prokaryotes (archaebacteria and eubacteria) and eukaryotes. If it is a common thought to consider prokaryotes more “primitive” than eukaryotes, it is therefore surprising to observe that prokaryotes use more recent and evolved regulatory molecules (proteins) whereas complex eukaryotes rather use the good old, and in a way more “primitive,” RNA networks for regulatory purposes (Figure 1).

### Regulatory Networks Are Stochastic

**FIGURE 2:**
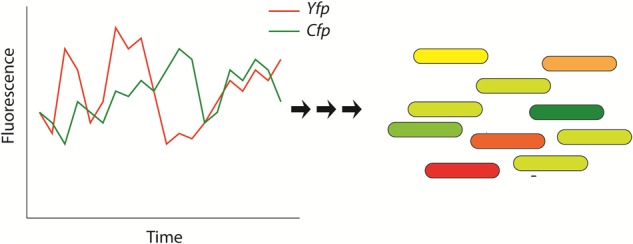
Schematic representation that illustrates the stochasticity of bacterial protein-based regulatory networks. This stochasticity is attributed to extrinsic and intrinsic noises and has been experimentally shown by measuring the fluorescence of bacteria that express two distinguishable fluorescent proteins: the Yellow Fluorescent Protein (YFP – shown in red) and the Cyan Fluorescent Protein (CFP – shown in green) (Elowitz et al., 2002). The genes of the fluorescent proteins are controlled by identical regulatory sequences (promoter). Cells that express the same amount of the two fluorescent proteins appear yellow, whereas cells exhibiting different quantities of fluorescent proteins will appear red or green. This figure has been adapted from the work published by Elowitz et al. (2002).

A particularly important property of genetic regulatory networks is their intrinsic “*stochasticity*” (Figure 2; Elowitz et al., 2002; Kaern et al., 2005; Losick and Desplan, 2008; Silva-Rocha and de Lorenzo, 2010; Locke et al., 2011; Ben-Jacob et al., 2014). This stochasticity in gene regulation has important impacts on the cell since the amount of many cellular components (DNA, regulatory molecules) is very low (Elowitz et al., 2002) and explains, for a large amount, the heterogeneity or cell-to-cell variations often observed in clonal populations of cells that are submitted to identical environmental/stress conditions. This observation has been well documented in various bacterial strains (e.g., *Bacillus subtilis, E. coli…*) (Ben-Jacob et al., 2014; Davis and Isberg, 2016) as well as complex eukaryotic organisms (Kar et al., 2009; Salari et al., 2012; Dacheux et al., 2017). One of the most famous and relevant single-cell experiments that explored stochastic gene expression is illustrated in Figure 2. In this study, Elowitz et al. (2002) analyzed the variability of expression of a specific promoter of *E. coli* (*E. coli*). The authors inserted two copies of this promoter in the genome of *E. coli*: one copy driving the expression of the Cyan Fluorescent Protein (CFP) and the second driving the expression the Yellow Fluorescent Protein (YFP). The authors reported high variability on the fluorescence type that was emitted by individual bacteria. The source of this variability relies on the stochasticity of genetic regulation that is explained by two different factors or noises (Elowitz et al., 2002; Silva-Rocha and de Lorenzo, 2010). First, “*extrinsic*” noises that arise because the expression of each gene/protein is controlled by the concentrations, fluctuations in the amounts, activities and locations of metabolites and regulatory molecules (e.g., polymerases, ribosomes…). Secondly, “*intrinsic*” noises imply that, even if the concentrations and states of every cellular component would be identical in every cell, the rate of expression of particular genes would also vary from cell to cell due to stochastic microscopic events that are intrinsic to transcription/translation events and influence gene regulation (e.g., collision rate) (Elowitz et al., 2002).

In prokaryotes and “simple” unicellular organisms, stochastic gene regulatory networks can be seen as advantageous since it allows sub-populations of bacteria to be prepared/conditioned to face and adapt very quickly to drastic environment changes (Schultz et al., 2009, 2013) and, therefore, can be considered as beneficial in various contexts: metabolism (ex: lactose utilization in *E. coli* (Ozbudak et al., 2004; Mettetal et al., 2006), stress response (ex: competence and sporulation in *B. subtilis* (Maamar et al., 2007), and pathogenesis (ex: antibiotic resistance in *Mycobacterium tuberculosis* (Stewart et al., 2003). On the other hand, it substantially limits the precision of gene regulation; which can be harmful. This is particularly true for complex eukaryotic organisms, in which the ultimate manifestations of this stochasticity can lead to aging (ex: murine cardiac myocytes (Bahar et al., 2006), cancers (Davies and Agus, 2015), neurodegenerative (ex: Alzheimer disease (Hadjichrysanthou et al., 2018), and autoimmune (Fatehi et al., 2018) disorders. Therefore, it seems that life, in particular in complex organisms, relies on a good compromise between randomness and determinism/finality. From randomness (stochasticity and trend to chaos of complex dynamic living systems) to the precise coordination of development, higher organisms have found a way to balance these two apparently opposite aspects of their internal way of working (Raj and van Oudenaarden, 2008).

### Complex Eukaryotes Require a Large Quantity of Non-coding RNAs for Their Regulation

As mentioned above, one of the main differences between prokaryotic and eukaryotic genomes is the proportion of non-protein coding DNA. Furthermore, this ncDNA portion increases with organism complexity: the ncDNA size follows a quadratic equation that is function of the total length of exonic DNA (Ahnert et al., 2008). Why do eukaryotes produce so much non-coding transcripts and how can this be correlated to their complexity? First, we have to consider that one of the main differences of complex eukaryotic organisms compared to prokaryotes is the spatio-temporal differentiation: with the specialization of cells into tissues, with all the different tissues that act together to coordinate organism homeostasis. Secondly, the generation time of complex eukaryotes can be much longer compared to prokaryotes or unicellular eukaryotes (examples: 20 min for *E. coli* (bacteria), 80 min for *Saccharomyces cerevisiae* (yeast) to several decades for human neurons). Since random events can be quantified by a frequency, organisms with longer lifetime are more prone to stochasticity and chaotic drift. Therefore, it is more obvious that eukaryotic cells need to undergo a much tighter regulation compared to prokaryotes since it is more crucial for eukaryotes to compensate and regulate randomness and better control cell fate.

How do eukaryotes satisfy this required tighter regulation? The answer most likely relies on the complexity of their regulatory networks. Indeed, recent studies have shown that more complex networks are better at coping with both: intrinsic and extrinsic noises that are the sources of stochasticity. Intrinsic noise tends to decrease with network complexity, and extrinsic noise tends to have less impact in complex regulatory networks (Cardelli et al., 2016). Nonetheless, the problem with protein-based regulatory networks is that their complexity is limited because of their restricted capacity to make interactions/regulations (Mattick and Gagen, 2005; Ahnert et al., 2008). Indeed, an interesting study conducted in yeast has revealed that the number of protein interfaces available for regulation or receiving regulation of other molecules is limited to 14 (Kim et al., 2006). With this restriction, various mathematical and theoretical studies have revealed that, given the genome sizes and the number of coding genes (proteins) found in complex eukaryotes, global regulation of all the genome components cannot possibly be achieved by proteins, since the number of regulations that proteins are able to do is way too low (Mattick, 2004; Taft et al., 2007; Ahnert et al., 2008). This is why it is very likely that the huge non-coding portions of eukaryotic genomes account for a large majority to the control of most genome components and regulate cell fate and homeostasis (Mattick, 2004; Pheasant and Mattick, 2007; Taft et al., 2007). In conclusion, ncRNAs are abundantly required in eukaryotes because they scale up the number of regulatory connections that is required for a fully integrated regulatory network (Ahnert et al., 2008).

Importantly, if the number of possibilities to receive/give regulations has not been established yet for RNAs, it is very likely that this number would be significantly higher compared to proteins. Indeed, proteins are more specialized molecules, whereas RNAs exhibit much higher plasticity in terms of structures, binding partners and therefore functions. For example, another interesting mechanism that RNAs could potentially use to increase the dynamic and plasticity of their high interaction potential is their possibility to interact with each other (*trans* interactions) and generate specific structural motifs that could modulate their interaction to binding partners (Doyle and Tenenbaum, 2014). Furthermore, in analogy to epigenetic for DNA, RNAs are also known to undergo diverse biochemical modifications. To date, well over hundreds of modifications were reported for RNAs (Grosjean, 2005). These modifications (with methylation predominating) can be added post-transcriptionally to every positions of either purine or pyrimidine rings (Cantara et al., 2011). Like epigenome, the, so-called, epitranscriptome is highly dynamic and include “writers” and “erasers” that modify coding or non-coding RNAs and “readers” that can translate these modifications into functional changes (Yang et al., 2018). Even if the functional relevance and molecular mechanisms of epitranscriptomic remain largely unexplored, it is very likely that these modifications shape RNA structure, stability and therefore adds many additional possibilities for regulating RNA interactions.

Interestingly, prokaryotes do not seem to require such a complex regulatory RNA network with only 12% of genomic ncDNA. Given their very short generation time and poor differentiation, it seems that harmful effects of stochasticity are limited. Instead, stochasticity is rather beneficial for them in order to adapt, respond and evolve much faster compared to complex eukaryotic organisms.

### Global Role of Regulatory RNA Networks

How can we integrate RNA- and protein-based regulatory networks from a global point of view? How are these two types of regulatory molecules linked to each other to control cell fate and decision? First, we have to consider that one of the main characteristics of RNAs, in contrast to proteins, is their high plasticity as mentioned above. Based on this property and what we discussed in this review, we postulate that one plausible global role of regulatory RNA ensembles/networks would be that using their high interaction/connection potential, they can “buffer” the stochasticity of genetic regulatory networks in order to guide cells toward an appropriate response observed upon specific stimuli and maintain homeostasis. According to this hypothesis, we might rather see regulatory RNAs as major “moderators” that supervise cellular pathways and guide cell decisions in order to prevent cells from chaotic drifts and death. This concept is interesting if we considered that primitive RNA networks led to the first life forms and remained conserved in modern cells. Consequently, RNA networks could be seen as the balance between randomness and determinism; it is therefore probably not surprising that life might have emerged from these RNA networks.

A recent study, conducted by Du et al. (2016), illustrates very well this hypothesis of RNA networks as regulators of stochasticity. In this study, the authors showed that an RNA regulatory network (composed of long-non-coding RNAs-lncRNAs) affects the expression of numerous protein-coding prostate cancer driver genes by acting as “sponges” by binding to miRNAs and preventing them to destabilize protein-coding transcripts. They demonstrated that this RNA network regulation is multiple: many protein-coding genes were regulated by only one lncRNA. Finally, they showed that restoring this lncRNA network is sufficient to suppress tumor activities in prostate cell lines. This study represents a good example where uncontrolled stochasticity (cancer) can be muted using an RNA network that acts as master regulator of several regulatory proteins/RNAs.

## Concluding Remarks and Future Perspectives

This view of RNA networks as master regulators that control the balance between randomness and determinism to control cell fate is important because it can change our perception of cell biology and provide new opportunities to design better therapeutic interventions. Indeed, we could imagine more efficient strategies to prevent or restore control on cancers, neurological disorders or senescence by focusing on these RNA regulatory networks rather than developing protein-based therapeutics (ex: antibodies, enzyme inhibitors…).

The emerging question is: how can we tackle the huge complexity of regulatory RNA networks given that we have only scratched the surface of protein-based networks? Nowadays, the current trend to fill in the gap of knowledge in the biology, functions and structures of regulatory RNAs is undoubtedly the use of high-throughput methods (Weidmann et al., 2016). Next-generation sequencing (NGS) is used for genome-wide measurements of inter- and intra-molecular RNA duplexes in living cells [PARIS, LIGR-Seq, and SPLASH (Aw et al., 2016; Lu et al., 2016; Sharma et al., 2016)] as well as for the identification of protein-binding partners [e.g., HITS-CLIP, PAR-CLIP (Zhang and Darnell, 2011; Li et al., 2014; Spitzer et al., 2014)], and several variants iCLIP, iCLAP (Huppertz et al., 2014; Li et al., 2014). If these methods have undoubtedly brought crucial information (big data) in the field of RNA, they didn’t really bring, so far, the expected breakthrough. This is probably because these data need to be used and transposed to detailed mechanisms of action in order to delineate general rules for regulatory RNA networks. In a similar manner to what we did with proteins, we need to use old school/traditional approaches (e.g., mutations to see effects on RNA structure and function) and study specific cases of regulatory RNAs with a particularly big emphasize on the structural data that currently cruelly lack.

### Fundamental Differences Between Proteins and RNAs and the Need of New and Adapted Approaches to Tackle the Complexity of RNA Networks

Whereas protein-based networks are highly specialized with delimited functions and tasks, RNA-based networks are much more complex systems to study. Indeed, RNAs are less specialized molecules that act as part of bigger networks where all individual RNAs can interact with many different other proteins and nucleic acids. RNAs also exhibit much more dynamic and plasticity in their structures, biochemical modifications (epitranscriptomic) and therefore binding partners and functions. In this regard, the recent progresses made in cryo-electronic microscopy (cryoEM) will certainly facilitate RNA structure determination and will also allow assessing the dynamic and plasticity of these structures.

In addition, this high functional plasticity of RNAs might explain why inactivation of a regulatory RNA can be trickier to analyze and will most likely generate more subtle changes with only partial destabilization of the downstream cell pathway(s). This is why we need to adapt our experimental approaches to the complexity of RNA networks. Therefore, the idea is to detect differences in the dispersion but not necessary in the average of the affected pathway(s). This is why single-cell analysis constitutes an attractive approach to highlight subtle changes in the cell (sub)-populations. In this approach, single cell are sorted and isolated using several well-established methods [e.g., micromanipulation, laser-capture microdissection, and fluorescence-activated cell sorting (Navin and Hicks, 2011; Hodzic, 2016)] as well as more recent techniques such as microfluidic (Yilmaz and Singh, 2012; Saliba et al., 2014). Then the use of high-throughput sequencing (whole-transcriptome analysis) on the isolated cells will allow amplification of small differences and will permit expression profiling and sequencing of coding and non-coding RNAs present in a single cell. Combined with strong statistical analysis, this offers the possibility to assess the transcriptomic heterogeneity and subtle changes occurring upon inactivation of a specific regulatory RNA. In addition, these high-throughput techniques can help us to identify the different RNA members of a network. Based on this identification, mutations of several members of the network can be envisaged to generate a detectable phenotype on the affected cell pathway.

### The Use of the Information Theories to Model the Complexity of RNA Networks

Finally, we need to better understand how the different regulatory RNAs work as networks and how they interact and are connected to each other as well as with DNA and regulatory proteins to better understand their roles in the cell biology.

How can we address this network complexity without considering the information theories? Indeed, a good analogy of complex biological regulatory network is the information network (Battail, 2013). In this analogy, all biochemical processes present in the cell can be seen as transfer of information. For example: (i) an hormone that binds to its receptor located on the cell surface to activate specific genes; and (ii) a population of bacteria that starts to sporulate under limiting nutrient conditions. These information transfers through the regulatory networks need to be robust and protected against the intrinsic and extrinsic noises, namely stochasticity, of the cell. It is surprising to note that information networks work exactly the same way since information transfer consists in sending “signals” through a network and the main purpose of this network is to protect these signals against noises until delivery point. In information theories, this protection of signals against noises is achieved using a network of, so-called, “correction codes” [ex: low density parity code (Wang et al., 2018), turbo codes (Valenti and Sun, 2002)]. These correction codes protect the original signals from noises in order to preserve the original content of these signals.

From this point of view, the analogy between RNA and information networks is striking. Since, as discussed in this review, we hypothesized that the major purpose of complex RNA networks such as the ones found in complex eukaryotic organisms is to “buffer” the stochasticity of biological systems. In other words, RNAs can protect living cells/organisms against chaotic drifts. With this view, RNA networks are analogous to “correction codes” found in the information theory. Therefore this involves that we could imagine to model RNA regulatory networks by extending information theories and thereby reach a wider and global comprehension of these molecules.

In conclusion, tighter regulation of complex organisms doesn’t rely on their genome size (number of nodes/genes), but rather relies on the possibility of each molecule (nodes) to receive or exert more regulations (edges). In higher and complex organisms, spatio-temporal regulation is crucial and needs to be more tightly regulated with much more noise control. This noise control network is probably strongly intricate with the catalyzers and lower level regulators from the biotic era. Life has adopted two strategies: either it diminishes the impact of noises by shortening the lifetime using fast replication (thanks to the efficiency of proteins) or it increases the noise control in complex organisms. As demonstrated by Ahnert et al. (2008) only RNAs can make a satisfactory number of possible edges compared to proteins and therefore create a sufficiently dense network for this purpose.

## Author Contributions

MV wrote most of the manuscript. MD participated to the manuscript writing. MV and MD contributed the most to the ideas presented in the manuscript. MD and MG gave regular intellectual inputs and proofread the manuscript.

## Conflict of Interest Statement

The authors declare that the research was conducted in the absence of any commercial or financial relationships that could be construed as a potential conflict of interest.
